# KCl-induced repetitive cortical spreading depression inhibiting trigeminal neuronal firing is mediated by 5-HT_1B/1D_ and opioid receptors

**DOI:** 10.1177/03331024221112998

**Published:** 2022-07-13

**Authors:** Weera Supronsinchai, Jan Hoffmann, Simon Akerman, Peter J Goadsby

**Affiliations:** 1 Department of Physiology, Faculty of Dentistry, Chulalongkorn University, Pathumwan, Bangkok, Thailand; 2 Headache Group, Institute of Psychiatry, Psychology and Neuroscience, King's College London, London, UK; 3 Department of Neural and Pain Sciences, University of Maryland Baltimore, Baltimore, Maryland, MD, USA; 4 Department of Neurology, University of California, Los Angeles, Los Angeles, CA, USA

**Keywords:** Cortical spreading depression, nucleus raphe magnus, serotonin, opioid, trigeminovascular system, migraine

## Abstract

**Background:**

We aimed to examine the effects of repetitive cortical spreading depression on the responses of nociceptive trigeminal neurons with dural afferents and characterize the role of 5-HT_1B/1D_ and opioid receptors.

**Methods:**

Trigeminocervical complex neurons (*n* = 53) responsive to nociceptive activation of the dura mater were studied in rats using electrophysiological techniques.

**Results:**

A sub-population (*n* = 32) showed an average inhibition of dural-evoked responses of 65 ± 14% from baseline with cortical spreading depression. This response was reversed by the selective 5-HT_1B/1D_ receptor antagonist, GR127935 (3 mg/kg; *n* = 6, iv), and a non-selective opioid receptor antagonist, naloxone (1.5 mg/kg; *n* = 6, iv), five minutes after injection. To determine the role of the nucleus raphe magnus in the trigeminocervical complex inhibitory effect, microinjection of lidocaine (2%, *n* = 6) or muscimol (100 mM, *n* = 5) into the nucleus raphe magnus was performed. There was no effect on cortical spreading depression-induced inhibition of neuronal firing in trigeminocervical complex by either.

**Conclusion:**

The data demonstrate that repetitive cortical spreading depression inhibits a subpopulation of dural nociceptive trigeminocervical neurons, an effect mediated by serotonin and opioid receptors. This inhibition does not involve modulation of nucleus raphe magnus neurons.

## Introduction

Migraine aura consists of a wave of cerebral hyperemia followed by oligemia (1). Physiologically, it is similar to cortical spreading depression (CSD) that is demonstrated in experimental animals and is believed to represent its experimental correlate, and thus serves as a useful pre-clinical model (2). CSD involves a wave of depolarization followed by suppression of cortical neuronal activity that moves across the cortex at a rate of 2–6 mm min^−1^ and is accompanied by cortical perfusion changes similar to those observed during aura (3–6). Experimental CSD can affect the trigeminovascular nociceptive system, specifically in rodents, causing neuronal activation in the trigeminocervical complex (TCC) and trigeminal ganglion (7,8) and inducing cortical vasodilation (9). Interestingly, it can inhibit trigeminal neurons when primary sensory cortex is involved (10). Furthermore, CSD can modulate activity in the nucleus raphe magnus (NRM), altering the processing of dural and facial trigeminovascular nociceptive information (11). CSD-induced trigeminovascular activation does not necessarily require a peripheral trigeminal input (11). Therefore, the mechanisms through which CSD can modulate nociceptive trigeminovascular activation are complex, and may involve brainstem structures (7,12,13).

The primary objective of our study was to determine the effects of repetitive CSD, using solid potassium chloride (KCl) application to the cortex, on dural-evoked nociceptive neuronal activation in the TCC. After observing an effect of KCl-induced CSD on dural-evoked nociceptive response, we then sought to determine its pharmacology using 5-HT_1B/1D_ and opioid receptor antagonists. Both receptor sub-types have been demonstrated to have an effect on TCC neuronal firing (14,15) and are involved in the descending pain modulating system (16,17). Furthermore, since activity in the NRM is known to be modulated by CSD, and NRM modulates dural and facial evoked nociceptive TCC activation, we examined the effects of local injection of lidocaine and the GABA_A_ receptor antagonist, muscimol into the NRM on the changes to TCC activity evoked by CSD. We hypothesized that CSD-induced changes of dural-evoked activity in the TCC may be reversed by 5-HT_1B/1D_ or opioid receptor antagonists, as well as altered by the descending control provided by the NRM.

## Experimental procedures

All experiments were conducted in accordance with a protocol authorized by the Institutional Animal Care and Use Committee of the University of California, San Francisco. The work conformed to the Guide for the Care and Use of Laboratory Animals produced by the National Institutes of Health, the guidelines of the Committee for Research and Ethical Issues of IASP (18) and the ARRIVE guidelines.

### Animal preparation

Male Sprague Dawley rats (250–350 g) were anesthetized with sodium pentobarbital (Nembutal; Lundbeck, Scottsdale, AZ) (60 mg kg^−1^ intraperitoneally). Withdrawal reflex after a paw pinch, and the corneal reflex were carefully observed to monitor the depth of anesthesia (19). A thermostatically controlled homeothermic blanket system was used to keep body temperature within a physiological range (TC-1000; CWE Ardmore, PA). The trachea was cannulated and rats were ventilated with oxygen-enriched air, 2–3 ml per stroke, 80–100 strokes min^−1^ (Model 683 small rodent ventilator; Harvard Instruments, Kent, UK). End tidal CO_2_ was monitored (Capstar 100; CWE) and kept between 3.5% and 4.5%. The femoral vein and artery were cannulated for intravenous (i.v.) anesthetic administration and arterial blood pressure monitoring (CT-1000; CWE), respectively. The rats were maintained under general anesthesia with propofol (25–35 mg kg^−1^ h^−1^ i.v.). The blood pressure, end tidal CO_2_, and temperature were electronically displayed for continuous monitoring. The head of the animal was fixed in a stereotaxic frame (Kopf Instruments; Tujunga, CA). Using an appropriate level of anesthesia, a midline cutaneous incision was made and the skull was exposed. A craniotomy was made above the parietal cortex using a saline-cooled dental burr to expose the middle meningeal artery (MMA). The muscles of the dorsal neck were separated and a C1 hemilaminectomy ipsilateral to the exposed MMA was performed to allow access to the trigeminocervical complex (TCC) for recording of trigeminal neurons.

### CSD induction

A midline incision was performed and a 2 mm craniotomy was made to expose the parietal cerebral cortex by the saline cooled drill technique. A portion of the dura mater was carefully removed using a needle to expose sufficient cortical surface for stimulation. CSD induction was achieved by gentle placement directly to the surface of the parietal cerebral cortex of 3 mg KCl that dissolved over 1–2 minutes, and confirmed with recording of direct current (DC) and cortical blood flow with laser Doppler flowmetry as described previously (20).

### Trigeminocervical complex activity recording

To record neuronal activity in the trigeminocervical complex (TCC), a tungsten recording electrode (1 MΩ; World Precision Instruments, Sarasota, FL) was lowered into the spinal cord in 5 μm steps using a piezoelectric motor–controller system (IW-811, Burleigh Instruments; 8200 Controller, EXFO, Plano, TX) (19). The signal from the recording electrode was connected to a high impedance head stage preamplifier (NL100AK; Neurolog, Digitimer, Herts, UK), fed via an AC preamplifier (Neurolog NL 104, gain ×1000) through filters (Neurolog NL125; bandwidth from 300 Hz to 10 kHz) and a 60 Hz line noise eliminator (Humbug; Quest Scientific, Vancouver, BC, Canada) to a second stage amplifier (Neurolog NL106) providing variable gain (×20 to ×30). This signal was fed to a gated amplitude discriminator (Neurolog NL201) and an analogue-to-digital converter (Power 1401plus; Cambridge Electronic Design, Cambridge, UK). The signal was processed (Spike 2 5.21, Cambridge Electronic Design, Cambridge, UK) and stored digitally. The filtered and amplified electrical signals from the action potentials were fed to a loudspeaker via a power amplifier (Neurolog NL120) for audio monitoring, and were displayed on analogue and digital storage oscilloscopes to assist isolation of the single unit activity from adjacent neuronal activity and noise.

Neurons in the TCC were identified by their response to ophthalmic division facial cutaneous receptive field stimulation and response to stimulation of trigeminal afferents that innervate the middle meningeal artery (MMA). Single units were recorded. Post-stimulus histograms (PSTHs) were established with trains of 20 stimuli. PSTH responses to electrical stimulation of the MMA afferents were recorded at 5 minute intervals to assess the baseline response. At least three baseline responses within a tolerance of 5% were collected to ensure that the neurons chosen were responding consistently and that there was no drift of the recording electrode. These baseline PSTHs were collected before KCl induction and PSTHs were further collected 2, 5, 10, 15, 20, 25, 30, 45, 60, 75 and 90 minutes after KCl induction. To activate trigeminal primary afferents, a bipolar stimulating electrode (NE 200; Rhodes Medical Instruments, Woodland Hills, CA) was placed on the dura mater with the poles either side of, or adjacent to, the MMA. Square wave pulses were used to stimulate afferents to the MMA at 0.5 Hz, 0.1–0.2 ms, and 10–15 V (S88 stimulator; Grass Instruments, West Warwick, RI).

### Post-recording processing

After completion of electrophysiology recording, the rats were euthanized with Euthasol (Virbac AH, Fort Worth, TX) (1 ml kg^−1^ i.v.), and the location of the recording site within the TCC was marked by a thermoelectrolytic lesion (anodal DC of 20 µA, 20 s). Brain and spinal cord were removed and fixed in 10% formalin. Sections from the spinal cord were stained with cresyl violet dye for identification of the recording site in the spinal cord.

### Pharmacological modulation of dural-evoked changes after CSD induction

The effects of CSD induction on dural stimulation-evoked neuronal responses in the TCC were dissected pharmacologically. All drugs for intravenous injection were dissolved in saline and dosed at a volume 1 ml/kg. The selective 5-HT_1B/1D_ receptor antagonist, GR127935 (21) (Tocris; Ellisville, MO) was injected at a dose of 3 mg kg^−1^. Naloxone hydrochloride, an opioid receptor antagonist (Tocris; Ellisville, MO), was injected at a dose of 1.5 mg kg^−1^.

### Microinjection into the nucleus raphe magnus

A burr hole was made in the skull over the cerebellar cortex to allow access to the nucleus raphe magnus (NRM) for microinjecting test substances and controls. Lidocaine solution (2% w/v; Hospira, Lake Forest, IL) was mixed with 2.5% (w/v) Chicago Sky Blue 6B for histological confirmation of injection placement. Only data collected from animals where the injection was verified were included in the reported analyses. Muscimol (100 mM), a GABA_A_ receptor agonist, was obtained from Tocris (Ellisville, MO), and made up in saline vehicle containing 2.5% (w/v) Chicago Sky Blue 6B. Both drugs were microinjected in 100 nL volumes using a Hamilton syringe (75 RN; Hamilton, Reno, NV) fitted with a 30G needle. The syringe was attached to a Kopf model 5000 microinjection unit (Kopf Instruments; Tujunga, CA) connected to a heavy-duty micromanipulator on a stereotaxic frame. The tip of the needle was inserted into the midline NRM (AP = –2.76 mm, D = –0.5 mm) according to coordinates from the atlas of Paxinos and Watson (22). 

#### Statistical Analysis

Using anatomical distance measurements between the trigeminal innervation of the dural meninges and the TCC in the medulla, and known nerve conduction velocities, the dural-evoked trigeminovascular responses were classified as Aδ-fibers (response 5–20 ms post stimulation) (23). The mean value of the baseline TCC neuronal firing was measured. The spontaneous background activity over 100 s was calculated and compared with mean spontaneous firing before and after KCl-evoked CSD. The results are expressed as a percentage of the mean value and the standard error of the mean (SEM) for each group. All responses were displayed and analyzed using Spike2 software (version 5, CED; Cambridge, UK). The critical ratio test (24) was used to determine if a unit was inhibited, which was considered as at least a 30% reduction in firing. A mixed model ANOVA for repeated measures was performed, applying Greenhouse–Geisser corrections if the assumption of sphericity was violated, to compare vehicle and treated groups. Post-hoc comparisons were made using *t*-tests for time point comparisons with a Bonferroni correction. Differences between groups were determined using independent *t*-tests. The number of CSDs in each group are count data and were compared using the Median test for the unaffected versus inhibited group, and the Kruskal-Wallis one-way analysis of variance across the inhibited cells. Statistical analysis was carried out using SPSS (version 19/26, Chicago, IL). Statistical significance was set at *P* < 0.05.

## Results

### Site of recording

**Figure 1.**
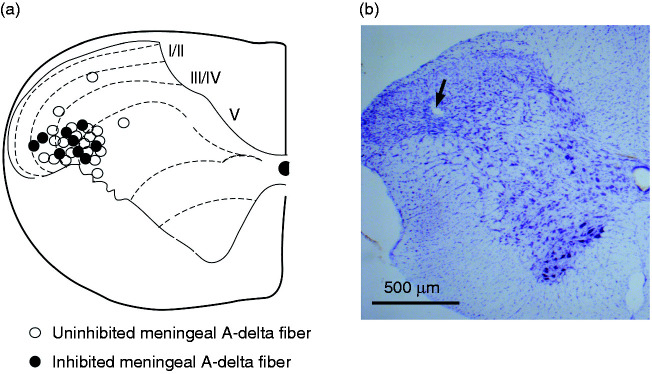
(a) The locations of recording site in the trigeminocervical complex (TCC) at the level of C1 responding to electrical stimulation of afferents from the middle meningeal artery, its branches, and periarterial dura as indicated by thermoelectrical lesions. The locations were reconstructed from unaffected trigeminal cell firing animals (closed circle) or from inhibitory trigeminal cell firing animals (open circles) and (b) Example of a thermoelectrical lesion site in the TCC (arrow).

Cortical application of KCl 3 mg evoked CSD on the ipsilateral side to the TCC recording site in all animals tested (*n* = 53 rats). Recordings were performed in the trigeminocervical complex (TCC) at the level of C1. A total of 53 neurons (*n* = 53 rats) responding to electrical stimulation of the dural-MMA were identified for analysis. The location of the recording sites of neurons in the TCC that responded to dural-evoked stimulation was the deep layers (laminae III to V) of the spinal dorsal horn of the TCC (Figure 1 A–B).

### Effects of KCl-induced CSD on TCC neuron firing and spontaneous background activity

#### Effect of KCl ipsilateral to the TCC

**Figure 2.**
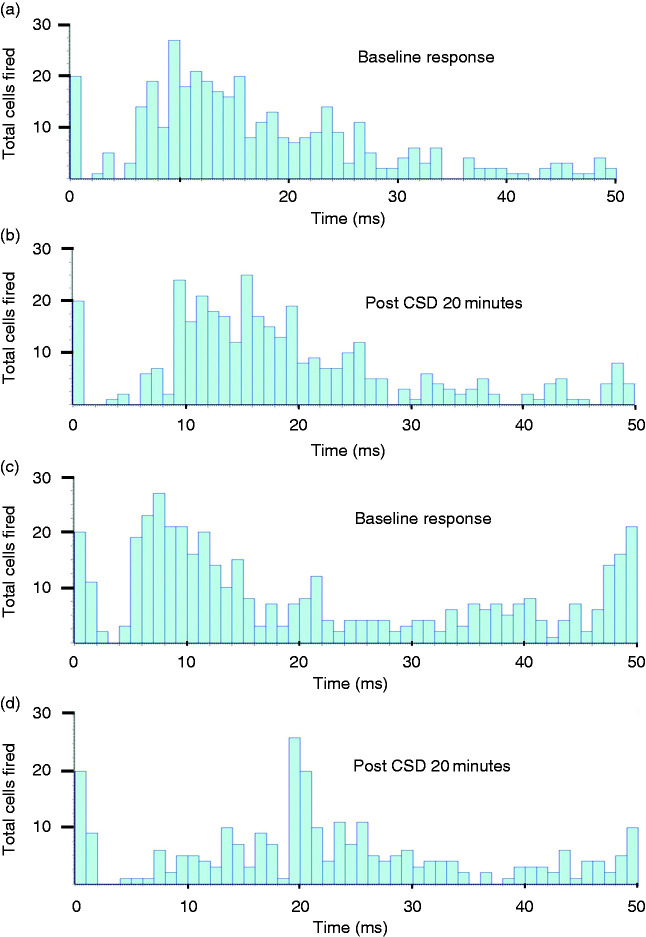
Post-stimulus histograms of neuronal responses to electrical stimulation of the dura mater around the middle meningeal artery (MMA) for two distinct outcomes A/B and C/D. (a) Baseline response before CSD; (b) 20 minutes after CSD. The units with A-fiber input are not affected by KCl induced CSD. (c) Baseline response before CSD and (d) 20 minutes after CSD. The units with A-fiber input are inhibited by KCl-induced CSD. Units firing over 20 sweeps of 50 ms are shown.

**Figure 3.**
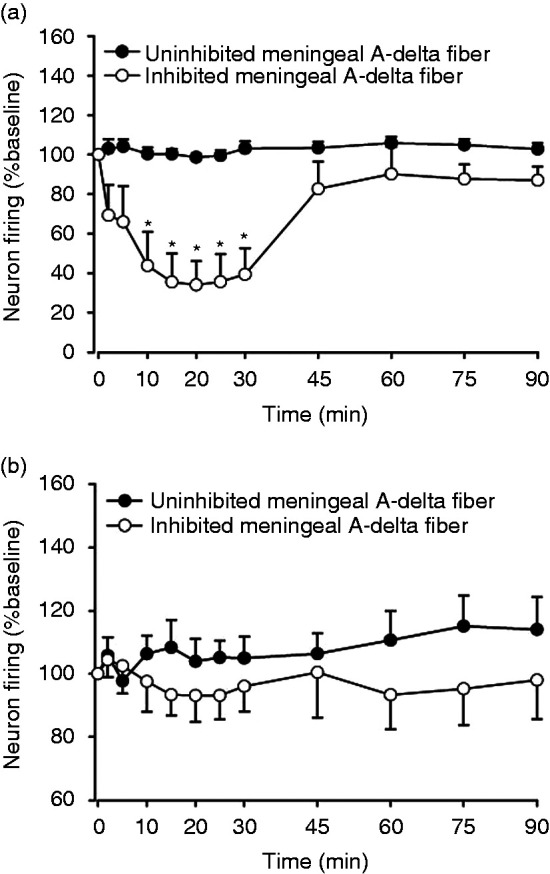
Effect of cortical spreading depression induced by 3 mg KCl on post-stimulated histogram and spontaneous background activity in response to electrical stimulation of afferents from the middle meningeal artery, its branches, and periarterial dura. (a) Total 30 neurons, nine out of the 30 neurons showed an average inhibition of TCC neuron firing from baseline and (b) The spontaneous background activity was unaltered. *P < 0.05 significance compared with baseline. Meningeal A-delta fiber refers to trigeminocervical neurons receiving input from a dural afferent.

Two different populations of trigeminal neurons responsive to nociceptive activation of the MMA, after KCl induction, are described. In twenty-one neurons (21 rats), the dural-evoked neuronal responses did not vary over 90 minutes when compared with baseline (*r*^2^ = 0.022, *P* = 0.646). However, dural-evoked responses in nine neurons (nine rats) were significantly inhibited, with a maximum effect of 65 ± 14% of baseline at 15 min after KCl induced CSD (*F*_2,20_ = 5.550, *P* = 0.009; Figure 2). Significant changes were seen from 10 min (*t*_8_ = 3.283, *P = *0.011) to 30 min (*t*_8_ = 4.570, *P* = 0.002; Figure 3A) with a gradual recovery of inhibition to baseline after KCl induction. Spontaneous background activity was not significantly different in all groups when compared with baseline in dural-evoked TCC neurons that were not inhibited, (*F*_2,51_ = 1.317, *P* = 0.277) and in those that were inhibited (*F*_2,19_ = 0.456, *P* = 0.677; Figure 3B).

#### Effects of intravenous injection of GR127935 or naloxone on inhibited dural-evoked TCC neurons

**Figure 4.**
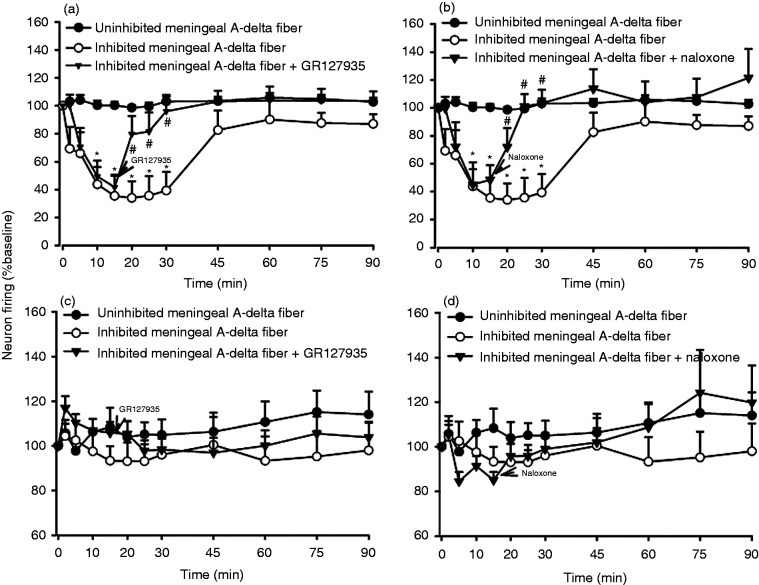
Effect of intravenous injection of GR127935 and naloxone on KCl-induced inhibition of trigeminal neuron firing in response to electrical stimulation of afferents from the middle meningeal artery, its branches, and periarterial dura. Intravenous injection of (a) GR127935 (3 mg/kg) and (b) naloxone (1.5 mg/kg) had significant reverse the inhibition of trigeminal neurons firing 5 minute after injection compared with inhibition of trigeminal neuron firing. (c) and (d) Spontaneous background activity had no significant different to baseline. *P < 0.05 significance compared with inhibitory trigeminal neuron firing. Meningeal A-delta fiber refers to trigeminocervical neurons receiving input from a dural afferent.

The 5-HT_1B/1D_ receptor antagonist, GR127935 (3 mg/kg, i.v., *n* = 6) or non-selective opioid receptor antagonist, naloxone (1.5 mg/kg, i.v., *n* = 6) were injected 15 min after CSD induction, in separate experiments, at the time of maximum inhibition of the TCC neuronal firing. GR127935 significantly antagonized the effects of CSD-induced inhibition of the dural-evoked neuronal responses 10 minutes after injection (GR127935, *t*_13_ = 2.504, *P* = 0.026; Figure 4A), while the effect of naloxone was seen at 20 minutes (*t*_13 = _3.471, *P* = 0.004, Figure 4B) respectively, when compared with inhibition of TCC neuronal firing in the control group. Spontaneous background activity was not significantly different to baseline (GR1279365, *F*_3,14_ = 2.673, *P* = 0.093, Figure 4C; naloxone, *F*_2,12_ = 1.829, *P* = 0.199, Figure 4D). Injection of vehicle (*n* = 9) had no effect on either spontaneous or evoked firing in the TCC.

### Effects of microinjection of lidocaine or muscimol into the NRM on inhibited dural-evoked TCC neurons

The sodium channel blocker, lidocaine (2%, 100 nL, *n* = 6) or GABA_A_ receptor antagonist, muscimol (100 mM, 100 nL, *n* = 5) were microinjected into the NRM 15 minutes, in separate experiments, after CSD-induced inhibition of dural-evoked TCC neuronal firing. Neither lidocaine nor muscimol were able to antagonize the effect of CSD-induced inhibition of neuronal firing in the TCC (lidocaine, *F*_1,7_ = 2.146, *P* = 0.188, Figure 4A; muscimol, *F*_2,9_ = 0.928, *P* = 0.443, Figure 4B) when compared with inhibition of TCC neuronal firing in the control group. Spontaneous background activity was not significantly different from baseline (lidocaine, *F*_1,7_ = 2.146, *P* = 0.188, Figure 4C; muscimol, *F*_2,9_ = 0.928, *P* = 0.443, Figure 4D).

### Effect of interventions on cortical spreading depression (CSDs)

Exposure of the cortex to KCl resulted in 15 (median, 12,17-interquartile range [IQR]) CSDs over 90 minutes in the group with no change in TCC firing; no different to the inhibited group (10, 7,12; χ_1_ = 2.3, *P* = 0.13). There were 17 (16,18) in the naloxone group, and 14 (11, 15) in the GR127935 treated groups. In the NRM injected groups there were 11 (11,12) in the muscimol group and 16 (12,17) in the lidocaine group. Across the inhibited neurons the naloxone group had more CSDs than the control group (χ_4_ = 13.1 for the Kruskall-Wallis test; *P* = 0.009 for the pair-wise comparison) with no difference in the other groups.

## Discussion

The data focus on a subgroup of neurons in the trigeminocervical complex responsive to dural peri-middle meningeal artery stimulation that are inhibited by repetitive CSDs induced by solid 3 mg of KCl topical application to the cerebral cortex. This inhibition can be reversed by intravenous injection of the 5-HT_1B/1D_ receptor antagonist, GR127935 and the opioid receptor antagonist, naloxone. The inhibition does not seem to involve the nucleus raphe magnus (NRM), since microinjection of lidocaine and muscimol into the NRM did not alter it.

Activation of different brain areas can modulate nociception. The cerebral cortex has been shown to modulate pain by acting on pronociceptive and anti-nociceptive circuits mediated by changes to GABAergic neurotransmission in the insular cortex. These changes can induce analgesia or hyperalgesia (25). This effect may be implicated in the mechanism of endogenous pain modulation, since KCl-induced CSD with microinjection into the different cortical areas has been shown to produce different responses on trigeminal meningeal-evoked cell firing. Microinjection of KCl into the insular cortex and primary sensory cortex induced facilitation and inhibition, respectively, of meningeal evoked response in Sp5C of trigeminal spinal cord without effects on cutaneous nociceptive responses (10).

CSD can be triggered by a range of stimuli: mechanical, electrical or chemical (5,26). It has been demonstrated that the different stimuli may initiate CSD through different mechanisms. Mechanical stimuli (pinprick) can evoke a single episode of CSD and have been shown to involve sodium ion channels (27), and an electrical stimulus involves at least glutamate N-methyl-D-aspartate (NDMA) receptors (28). KCl evokes multiple CSDs with a range of transmitters being involved in its mediation (29). These data may imply that CSD can be induced by more than one pathophysiological mechanism, and these may be involved in the pathogenesis of migraine aura and may explain the different therapeutic approaches in migraine patients.

In previous studies CSD has been shown to activate trigeminocervical (TCC) neurons via peripheral meningeal nociceptors (9). Whether there is a central pathway, or involvement, for the TCC activation has been the subject of some discussion. Zhang and colleagues (12) demonstrated that CSD increased spontaneous background activity in trigeminal ganglion, and similarly in central trigeminal neurons. In contrast, lidocaine microinjection into trigeminal ganglion, to remove the peripheral trigeminal afferent input, had no effect on CSD-induced spontaneous background activity (13). The effect of CSD induced by pinprick, measured by cortical hyperemia, is without change of trigeminal basal discharge rate or superior sagittal sinus stimulation-evoked response in the TCC neurons in cats (30). Our data are consistent with those findings, although do not reconcile the differences with other studies. Taken together, it could be suggested that CSD effects on TCC neurons could be both “top-down” and “bottom-up”, resulting in a complex pathophysiology. Interestingly, activation may be generated within the brain or specifically in brain areas, including the brainstem (periaqueductal gray (PAG) and NRM) that are connected to the cortex (11), and the thalamus (31). Our new data show that CSD has a strong inhibitory effect on about one-third of TCC neurons. This inhibition can be reversed by intravenous injection of a 5-HT_1B/1D_ receptor antagonist and an opioid receptor antagonist which makes a desensitization mechanism to account for the findings unlikely. Microinjection of lidocaine and muscimol into the NRM had no effect on this inhibition, implying that it has no significant role in any centrally mediated inhibitory process.

**Figure 5.**
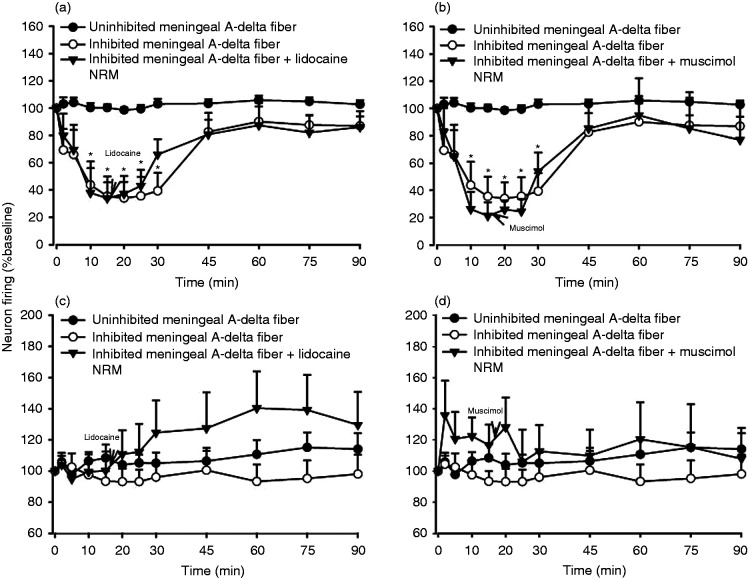
Effect of microinjection of lidocaine and muscimol into the NRM on KCl-induced inhibition of trigeminal neuron firing in response to electrical stimulation of afferents from the middle meningeal artery, its branches, and periarterial dura. (a) This inhibition was unaffected by microinjection of (a) lidocaine (2%, 100 nl) and (b) muscimol (100 mM, 100 nl) into the NRM. *P < 0.05 significance compared with  inhibitory trigeminal neuron firing. (c) and (d) Spontaneous background activity had no significant different to baseline. **^#^***P* < 0.05 significance compared baseline. ^#^*P* < 0.05 significance compared with unaffected trigeminal neuron firing. Meningeal A-delta fiber refers to trigeminocervical neurons receiving input from a dural afferent.

The PAG and the NRM have been shown to receive descending inputs from the cortex (32) and project to the trigeminocervical complex (33). The neurotransmitters that are involved in this projection from cortex to PAG and NRM remain unknown. The NRM contains serotonergic neurons that project to the spinal dorsal horn of spinal cord and the trigeminal nucleus caudalis. Stimulation of neurons in the NRM inhibits trigeminovascular neuronal response to dural mechanical stimulation. In one study, several neurons showed antagonism of the lidocaine –induced inhibition of trigeminovascular responses to dural mechanical stimulation by induction of CSD with KCl in the cerebral cortex, while some showed no response to the trigeminal inhibition (13). The cortical activation evoked by CSD would feed downward to alter PAG and NRM output, and reduce the discharge rate of these neurons. This in turn would reduce the descending inhibition to the trigeminal nucleus caudalis resulting in increased activation.

The serotonergic and opioidergic systems are important in modulating descending nociceptive projection to the spinal dorsal horn and potentially the trigeminal nucleus caudalis. 5-HT receptor subtypes, including 5-HT_1A_, 5-HT_1B_, and 5-HT_1D_, are located in PAG and NRM. Intravenous administration of zolmitriptan (34) or naratriptan (35) can inhibit the TCC neuronal firing induced by dural stimulation. Naratriptan microinjection into the vlPAG decreases TCC neuronal firing to electrical stimulation of the dura mater but not facial stimulation (16). Similarly, nociceptive trigeminovascular thalamic neurons in the ventroposteriormedial nucleus (VPM) activated by stimulation of the superior saggital sinus can be locally inhibited by microiontophoresis of naratriptan (36). Co-injection of naratriptan and the 5-HT_1B/1D_ receptor antagonist, GR127935, inhibits this effect (36). Taken together the data indicate multiple plausible sites of action for triptans, 5-HT_1B/1D_ receptor agonists.

## Conclusion

The data demonstrate that repetitive cortical spreading depressions (CSDs) inhibit a subpopulation of dural nociceptive trigeminocervical neurons, an affect mediated by 5-HT_1B/1D_ and opioid receptors. There is no clear role for the nucleus raphe magnus in this inhibition. The data illustrate some part of the complexity of CSD interaction with trigeminal mechanisms, which is likely only one part of the overall pathophysiology, with ascending and descending mechanisms combining in the neurobiology of these phenomena. Understanding how the cerebral cortex modulates trigeminovascular nociception will improve our understanding of the pathophysiology of migraine, including the potential transmitters that can be manipulated therapeutically.

## Article highlights

- Cortical spreading depression (CSD) elicited by KCl application inhibits a sub-population of nociceptive trigeminocervical neurons.
- The inhibition of nociceptive trigeminocervical neurons after CSD induction can be reversed by serotonin 5-HT_1B/1D_ receptor and non-specific opioid receptor blockade.
- CSD elicited inhibition of nociceptive trigeminocervical neurons does not involve the nucleus raphe magnus.
